# First Description of the Underwater Sounds in the Mediterranean Monk Seal *Monachus monachus* in Greece: Towards Establishing a Vocal Repertoire

**DOI:** 10.3390/ani13061048

**Published:** 2023-03-14

**Authors:** Isabelle Charrier, Chloé Huetz, Léa Prevost, Panagiotis Dendrinos, Alexandros A. Karamanlidis

**Affiliations:** 1 Institut des Neurosciences Paris-Saclay, Université Paris-Saclay, Centre National de la Recherche Scientifique, UMR 9197, 91400 Saclay, France; 2 MOm/Hellenic Society for the Study and Protection of the Monk Seal, Solomou Str. 18, 10682 Athens, Greece

**Keywords:** acoustic monitoring, anthropogenic noise, conservation, Mediterranean monk seal, Pinnipedia, underwater vocalization

## Abstract

**Simple Summary:**

We describe for the first time the underwater sounds of the Mediterranean monk seal; the study was carried out during the 2021 pupping season in an important reproductive area of the species in Greece. We defined 18 different call types included in three main categories: harmonic, noisy and pulsative calls. We also described the soundscape of the study area to highlight the anthropogenic disturbance this endangered marine mammal faces in its natural environment.

**Abstract:**

The Mediterranean monk seal *Monachus monachus* is one of the most endangered pinnipeds in the world, and is classified as “Endangered” by the International Union for the Conservation of Nature. Any additional knowledge about the species is invaluable to its effective conservation. In the present study, we deployed an autonomous underwater recorder in an important reproductive area of the Mediterranean monk seal in Greece to describe its underwater vocal repertoire. Over the 330 h of continuous recordings, 9231 vocalizations were labelled as potentially produced by Mediterranean monk seals, and 1694 good quality calls were analyzed. We defined 18 call types divided into three main call categories: harmonic, noisy, and pulsative calls. We also described the soundscape in which this endangered species lives and found that human activities around the two main pupping caves had a strong impact on the sonic environment of these seals: the noise level produced by boat traffic was high, and occurred on an hourly (25 to 50 min/hour) and daily basis (10.8 to 16.9 h/day). Such high levels of noise might not only impair the communication of the species, but also impact its survival, as chronic noise can induce physiological stress.

## 1. Introduction

The Mediterranean monk seal *Monachus monachus* is classified by the International Union for the Conservation of Nature (IUCN) as “Endangered” [1], and is considered to be one of the most endangered pinnipeds in the world, with a global population of approximately 1000 individuals [2]. Following centuries of continuous declines that led the species to the brink of extinction, concerted conservation efforts have now managed to reverse the tide, and the species is slowly recovering. In the eastern Mediterranean Sea, one of the species’ main strongholds, Mediterranean monk seals have recently increased their range in Greece [3] and recolonized Cyprus [4] and Albania [2]. However, in a highly human-dominated and rapidly changing environment, such as the one in the eastern Mediterranean Sea, the conservation of the species remains a real challenge. To better protect the Mediterranean monk seal, any additional knowledge on its biology, ecology, and behavior is an asset that will contribute towards the development and implementation of more effective and management actions [5].

Vocal communication in pinnipeds is highly developed and acoustic signals are of vital importance in many basic biological functions, such as breeding, territorial defense, maternal care, and predation avoidance [6,7]. Important information about the emitter can be assessed from its vocalizations, including individual identity, age/class, sex, motivation, body condition, or social rank [8]. In some cases, the description of the vocal repertoire of a given species can be very useful in detecting its (seasonal) presence. Such acoustic monitoring is commonly used in marine species, such as polar phocids [9,10,11,12] and cetaceans [13,14,15]. Acoustic monitoring is particularly important in elusive species (e.g., blue whales *Balaenoptera musculus* and beaked whales *Ziphiidae* family) [16,17,18], in which visual surveys are often difficult to perform because of irregular breathing intervals and/or short surface times. As sound travels faster in water than in air, underwater passive acoustic monitoring has been frequently used during the last decades in marine mammal research, as it allows for long-term monitoring (i.e., over a year) over large areas. Detection of vocalizations can be also efficient over very long distances, especially for species producing low-frequency calls, such as baleen whales (i.e., *Mysticeti* family) [16,19,20]. When the vocal repertoire of a given species has been described, automatic detection algorithms can be used to analyze (long-term) acoustic recordings and assess the presence of a given species in an area of interest, and thus acquire knowledge on the seasonal and temporal activity patterns/presence [12,18]. Passive acoustic monitoring can also give important information on the sonic environment of a study species; of particular interest are the levels of anthropogenic noise, as it can have a strong impact on marine biodiversity. Noise occurrence in oceans has dramatically increased since the industrial age, with increasing shipping activity resulting in an estimated 32-fold increase in low frequency noise over the last 50 years [21]. Anthropogenic noise can impact all forms of marine life, from fishes and invertebrates [22] to marine predators, such as cetaceans [23] and seals [24], and diving and flying seabirds [25,26]. Reported impacts range from fatal strandings, hearing damage (temporary or permanent), avoidance of noisy areas, change and interference in behaviors (e.g., foraging, breeding, socializing) to vocal/hearing masking [23,27]. Assessing anthropogenic noise levels is therefore crucial to better assess the threat marine species are exposed to, and thus to develop effective mitigation and conservation plans.

The aerial vocal repertoire of the Mediterranean monk seal has been studied only recently [28,29], and five call types have been described so far: bark, chirp, grunt, short scream, and scream. As in most phocids, male Mediterranean monk seals have been recorded in the Cabo Blanco Peninsula (Ras Nouadhibou) defending aquatic territories in the proximity of important pupping caves [30]. In the eastern Mediterranean monk seal population, and in Greece in particular, aggressive interactions between adult males defending aquatic territories have not been observed directly, but have been witnessed indirectly by the records of injuries and scares on their flippers and neck [2,31]. It is therefore assumed that such aggressive behavior occurs in this part of the current species’ range. As in other phocids, male Mediterranean monk seals are therefore likely to produce underwater vocalizations to defend their territory while interacting with competitors or with potential mates [7]. A captive male Hawaiian monk seal (*Neomonachus schauinslandi*) has been recorded producing underwater vocalizations during, but also outside the breeding season [32].

As no information is currently available on the underwater vocal repertoire of the Mediterranean monk seal, we developed a research project aiming to study the underwater vocalizations of the species. Such findings will help in developing new monitoring methods for the effective conservation of this endangered species. In order to maximize our chances of collecting data, while minimizing disturbance at the same time, we deployed an autonomous underwater recorder and monitored an important reproductive area of the Mediterranean monk seal in Greece. Up to now, anthropogenic noise has not been investigated in key natural habitats of the Mediterranean monk seal. In the study area intense human activity was observed, and we therefore assessed the noise levels generated by these anthropogenic activities and described the soundscape in which this endangered marine mammal lives.

## 2. Materials and Methods

### 2.1. Study Site

The underwater vocal repertoire of the Mediterranean monk seal was studied for 15 consecutive days (18 November–2 December 2021) at northern Evia Island; this time period falls within the pupping period of the species [33]. Previous research in the study area identified two important pupping caves and an open beach that have been used in recent years by mothers and pups for resting [34] and a minimum of twelve different animals of all age and sex classes [2]. The study area was located in an area of intense human activity, including an aquaculture installation and a busy shipping lane less than 1 nautical mile from the pupping caves.

### 2.2. Acoustic Recording and Analysis

After centuries of persecution, Mediterranean monk seals in the eastern Mediterranean Sea are extremely wary of humans and are therefore extremely difficult to approach and study [35]; we therefore decided to use passive, non-invasive methodologies in our study. A passive acoustic monitoring device (UW-H100, Smiot, Toulon, France) was thus deployed at approximately 5 m depth, anchored at the sea floor. The device was placed at a distance of approximately 50 m to the west of the entrance of an important pupping cave and approximately 200 m to the east of an open beach; both were used for resting and nursing by adult females and their pups during the study period [36]. The hydrophone used was a SQ26 (Sensor Technology; Linear Frequency Range: ±1 dB, 0.001 to 28 kHz, device sensitivity: −168.5 dB re V/μPa). Continuous recordings were performed at a sampling frequency of 22.050 Hz.

To facilitate the analysis, the sound files of each recording day were merged to a single 24-h file and were subsampled at 11.025 Hz to visualize the vocalizations on spectrograms.

We labelled and analyzed all vocalizations using Avisoft SASLab Pro (Avisoft Bioacoustics, Nordbahn, Germany) and produced spectrograms using 1024-point Fast Fourier Transform, 75% overlap, and Hamming window. Clearly audible vocalizations were aurally and visually identified and classified. Call types qualitatively similar to the in-air call types described for the Mediterranean monk seal [28], and to the underwater call types of the Hawaiian monk seal [32] were given the same name. The names of new call types were assigned onomatopoeically, as the behavioral context or the biological function of the new call types could not be determined from the monitoring system used.

Based on their main acoustic structure, we categorized the different call types into three main categories: harmonic, noisy, and pulsative calls. Harmonic calls showed a clear harmonic structure with a fundamental frequency and a series of harmonics. In some cases, some harmonic calls were degraded (i.e., only the lowest harmonics were visible on the spectrogram) and consisted of only one or two frequency bands (i.e., fundamental frequency only or the fundamental frequency and one harmonic). Noisy calls were broadband calls without a clear harmonic structure and pulsative calls were either calls consisting of repetitive units produced at a constant rate or continuous calls with a fast amplitude modulation.

Representative spectrograms (Hamming window, FFT window size: 512 pts, 90% overlap) for each call type were generated using the Seewave package [37] in RStudio v 1.2.5042 software [38].

Depending on the call category, different acoustic features were measured in both temporal and frequency domains. Only good-quality calls that did not overlap with any other noise or vocalization were analyzed.

For harmonic calls, we measured, whenever possible, seven acoustic variables: dur, f0, Fmax, Q25, Q50, Q75, and excF. Total duration (dur, s) was assessed from the oscillogram. All frequency parameters were measured from the average energy spectrum: the fundamental frequency values (f0, Hz) were measured using the harmonic cursor, whereas the highest energy peak (Fmax, Hz) and the various energy quartiles (Q25, Q50, Q75, in Hz) were measured using the spectral characteristics function of Avisoft SAS Lab Pro. When possible, we also calculated the excursion frequency (excF, in Hz) on the first visible frequency band by measuring the absolute difference between the higher and lower frequencies on the spectrogram. For call types that showed only one or two frequency bands, we did not compute the energy quartiles.

For noisy calls, five acoustic variables were measured: duration (dur), the highest energy peak (Fmax), and the three energy quartiles (Q25, Q50, and Q75).

For pulsative calls, and depending on the call type, two to six variables were measured: duration (dur), the highest energy peak (Fmax), the three energy quartiles (Q25, Q50, and Q75), and the pulse rate (PR, in Hz). Depending on the call type, the pulse rate was measured either by using the Pulse Train Function analysis in Avisoft SASLab Pro (R. Specht, Avisoft Bioacoustics, Berlin, Germany) or by measuring the number of pulses per second on the oscillogram.

As our recordings were quite noisy (i.e., sounds from snapping shrimps from 1.5 to 10 kHz), we limited our spectral analysis between 20 and 1 kHz. This did not affect our results, as most call types showed most energy below 1 kHz. However, for two call types (i.e., growl and knock) the spectral analysis was performed between 20 Hz and 3 kHz, as these calls had energy above 1 kHz and were more powerful compared to the noise from the snapping shrimps. Screams were high-pitched calls and therefore the spectral analysis was performed between 20 Hz and 2 kHz.

In addition to the biotic sounds, our recordings included various sounds from boats; we labelled these sound events and calculated the total duration of “noise” per recording day (i.e., within 24 h) and the average noise duration per hour to assess whether the anthropogenic noise had a temporal pattern.

Finally, to describe and assess spectro-temporal changes of the soundscape in which Mediterranean monk seals live, we performed a Long-Term Spectrogram Average analysis (LTSA) of the 15-day study period. We used Triton and the remora soundscape-metrics https://github.com/MarineBioAcousticsRC/Triton/tree/master/Remoras/Soundscape-Metrics; accessed on 1 September 2021) to compute the following metrics: Power Spectrum Density (PSD), broadband (BB), and octave levels (OL).

### 2.3. Statistical Analysis

To validate the aural–visual classification of our call types, we carried out a Random Forest (RF) analysis [39,40] using the randomForest package in R [41]. We calculated the global accuracy of the RF classification (defined as accuracy = 1 − OOB, where OOB stands for out-of-bag error, i.e., the misclassification error rate) and computed the Gini index, which indicates the importance of each respective acoustic variable used in the classification. We included only acoustic variables that were measurable for all call types of a given category (6, 5, and 2 variables for harmonic, noisy, and pulsative calls, respectively). The number of variables randomly selected at each split was set at 2 for both harmonic and noisy calls, and at 1 for pulsative calls. The number of trees grown was set at 500.

## 3. Results

Over the 330 h of continuous recordings, 9231 vocalizations were labelled as potentially produced by Mediterranean monk seals. Due to the high levels of ambient noise (i.e., motorboat and snapping shrimps) and/or overlap with other vocalizations, only 1694 were analyzed. During the recording period, vocalizations were detected throughout the day. The time periods with the lowest number of detected calls were between 04:00 and 07:00 and 11:00 and 13:00 (range: 144 to 229 calls per hour cumulated over the 15 recording days).

### 3.1. Call Types

Based on the aural and visual characteristics from the spectrograms, we identified 18 different call types in total (i.e., harmonic calls *n* = 12, noise calls *n* = 3, pulsative calls *n* = 3).

**Figure 1:**
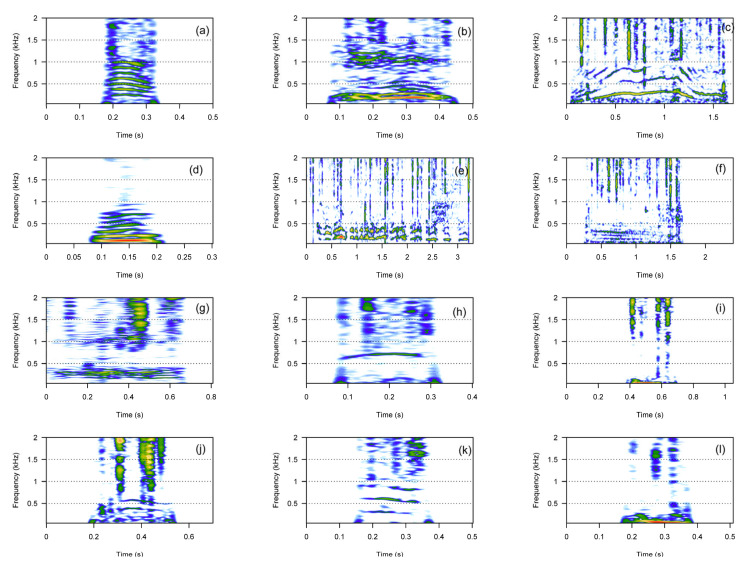
Spectrograms of the 12 harmonic call types. Bark (**a**), croak (**b**), cry (**c**), gloo (**d**), gloogloo (**e**), groan (**f**), moan (**g**), scream (**h**), whine (**i**), whoo (**j**), wop (**k**), wom (**l**).

For the category of harmonic calls, we described 12 call types: bark, croak, cry, gloo, gloogloo, groan, moan, scream, whines, whoo, wop, and wom (Figure 1). We analyzed 11 call types, as moans were too faint to be measured (Table 1 and Table S1). For some call types, such as screams, whines, and whoos, only one or two frequency bands were visible on the spectrograms. Moans were too faint to be measured reliably. All harmonic calls showed some frequency modulation pattern, and therefore the frequency excursion on the fundamental frequency could be measured. Most harmonic calls were short in duration (i.e., <0.5 s, Table 1), except whines, gloogloos, and cries, which showed average durations greater than 0.5 s. Most harmonic calls were low-pitched calls with a fundamental frequency below 200 Hz, except cries, screams, whoos, and wops. Screams were composed of a single frequency and showed the highest pitch with f0 values on average >1200 Hz (Table 1).

**Figure 2:**
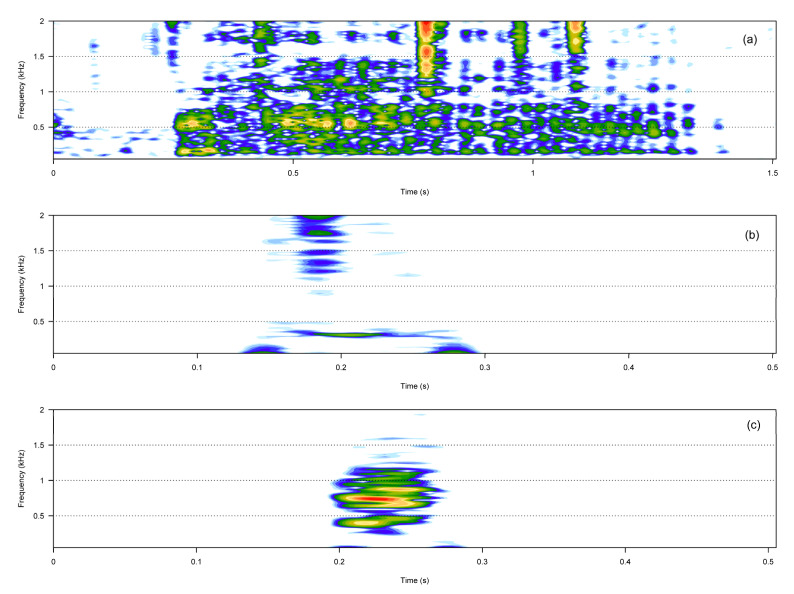
Spectrograms of the three noisy call types. Growl (**a**), hiccup (**b**), squeak (**c**).

The noisy call category included three call types: hiccups, squeaks, and growls (Table 1, Figure 2). Compared to hiccups and squeaks, growls were much longer and showed more energy in higher frequencies.

**Figure 3:**
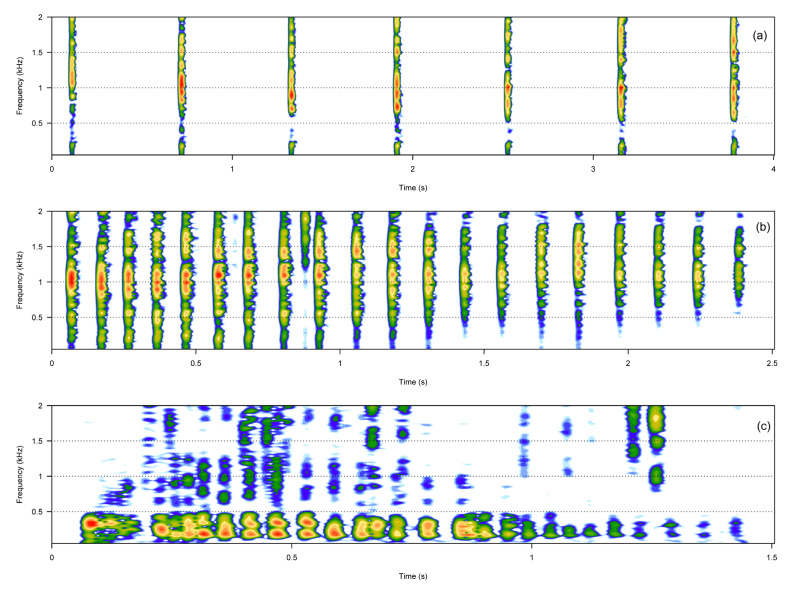
Spectrograms of the three pulsative call types. Clap (**a**), knock (**b**), rumble (**c**).

The pulsative call category included three call types: rumbles, knocks, and claps (Table 1, Figure 3). Rumbles were fast amplitude-modulated calls, whereas claps and knocks were composed of several short sound units that were always produced in series and at a constant rate. Knocks and claps were broadband calls, and, compared to all the other call types described, were often very loud.

The results of our RF analysis indicated that harmonic calls had a global accuracy of prediction of 79.3% for classifying the calls into the 11 call types (OOB error rate = 20.7%, Table 2), thus validating our aural–visual classification. The most important acoustic variables for the classification were f0, Q25, and Q75 (Gini index: 126.8, 99.6, and 75.96 respectively). For all 11 call types, correct classification rates (1-OOB) were greater than those obtained by chance (number of calls/total number of calls). The three call types showing the highest error rates were croaks, cries, and gloogloos. Croaks and cries were often confused with barks, and gloogloos with groans.

For noisy calls, the RF analysis indicated a global prediction accuracy of 95.4% (OOB error rate = 4.6%, Table 3). The most important acoustic variables for the classification were dur, Q75, and Q50 (Gini index: 189.5, 54.4, and 45.7, respectively). For all three call types, the correct classification rates (1-OOB) were greater than those obtained by chance. Only squeaks had a high error rate and were mostly confused with hiccups.

Finally, for pulsative calls, the RF analysis indicated a global prediction accuracy of 98.5% for classifying the calls into the three call types (OOB error rate = 1.54%, Table 4). The PR was the most important acoustic variable for the classification (Gini index: 53.5 for PR and 10.4 for Dur).

### 3.2. Boat Noise

Over the 15 days of continuous recordings, we found that the average hourly presence of boat noise ranged between 0.43 to 0.84 h, indicating that there was always some boat noise in our recordings. Boat noise was detected between 10.8–16.9 h per day. Some days were a bit quieter than others; only three days showed less than 12 h of noise per day (i.e., the 23^rd^, 27^th^, and 30^th^ November 2021).

### 3.3. Soundscape Analysis

**Figure 4:**
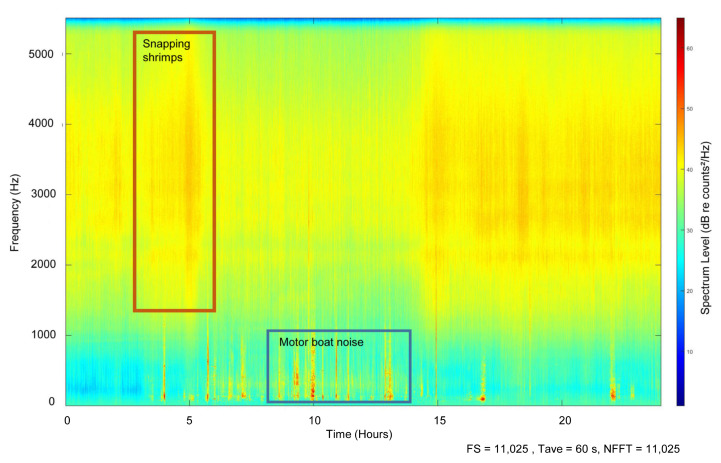
Example of a long-term spectrogram average of a 24-hours day of continuous recording (19 November 2021). The background “noise” generated by snapping shrimps (i.e., 1500–5000 Hz, red rectangle) and the “noise” generated by boat traffic (blue rectangle) present in the study area are presented. Monk seal underwater vocalizations can not be identified on the LTSA, as they are very short and masked by the vessel noise.

The LTSA soundscape analysis enabled the study of the sounds produced by human activity in the area (anthropophony, 10–1000 Hz), but also the study of the biotic sounds (biophony) generated by other species, such as snapping shrimps (i.e., *Alpheidae* family, 1.5 to 5.5 kHz) (Figure 4). The LTSA analysis enabled better visualization of the soundscape of the natural environment of the Mediterranean monk seal: this was dominated primarily by the sounds generated by the snapping shrimps (red rectangle) and the sounds of boats passing by (blue rectangle). These two “noise” sources masked the vocalizations of the seals in the area, which were recorded in the 50–1000 Hz frequency bandwidth.

**Figure 5:**
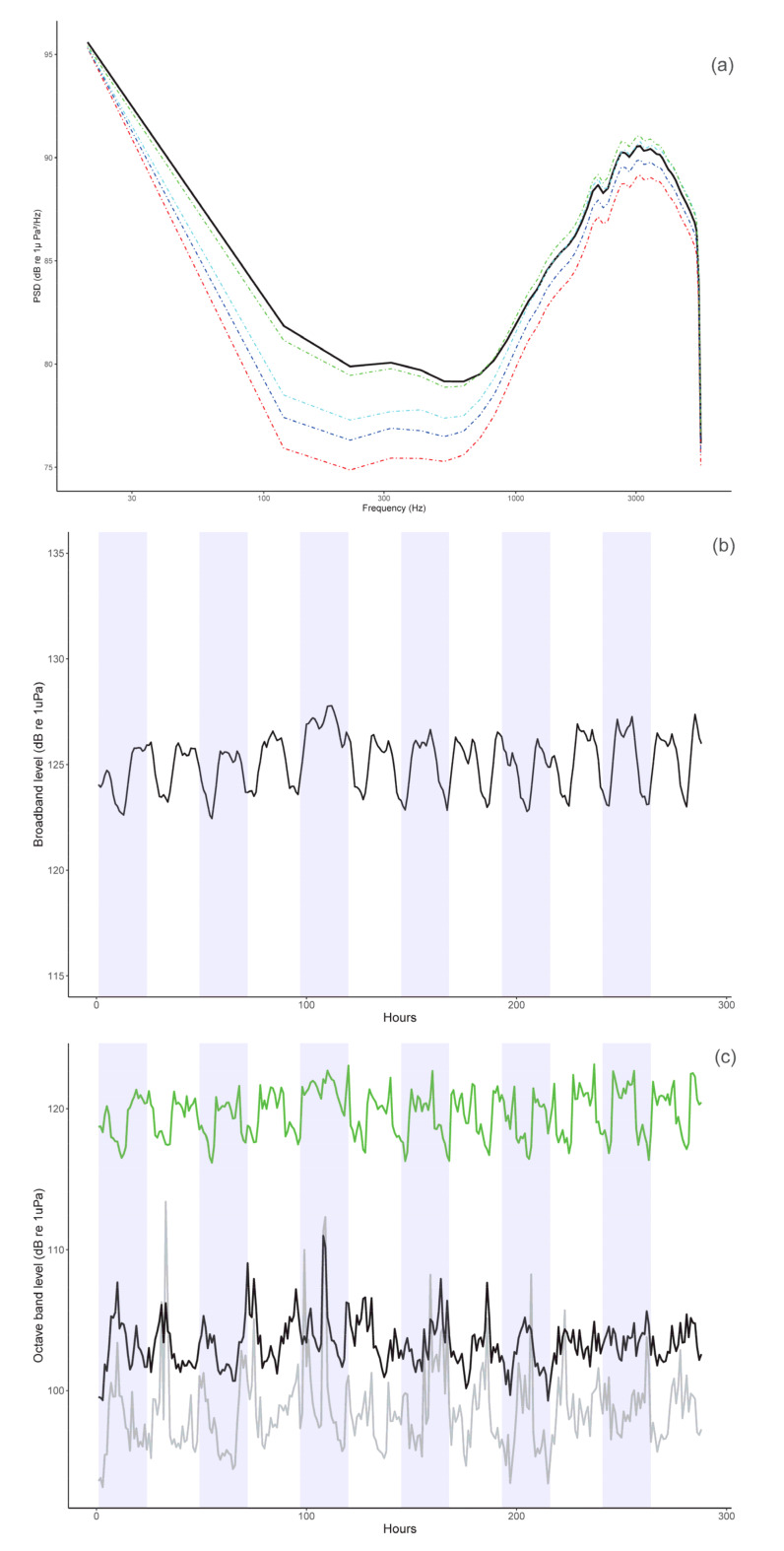
Power Spectrum Density (PSD, (**a**)), Broadband (BB, (**b**)), and Octave band level (OL, (**c**)) plots. Plots were computed over the 13 days of continuous recordings (19 November–1 December 2021). For the PSD plot, the median values are presented in black and the 10, 25, 75, and 90 percentiles in red, blue, cyan, and green, respectively. For the BB plot, the median is presented in black, and the white and blue background patterns indicate the 24-hours periods (i.e., a full day of recording). For the OL plot, we selected three representative frequency bands: the 125 Hz-band (grey) that represented boat “noise”, the 500 Hz-band (black) in which monk seal vocalizations were recorded, and the 2000 Hz-band (green) in which the snapping shrimps dominated.

Boat “noise” in the study area was present during all recording days and at least 10 h per day. Snapping shrimps were active almost all day long, but their sonic activity was often lower between 6 am to 2 pm. Their sound activity was not consistent during the study period (Figure 5b,c).

The PSD analysis (Figure 5a) indicated that noise generated by flow noise (mostly frequencies < 30 Hz) and boat traffic (20–200 Hz), as well as the noise generated by snapping shrimps (1.5–5 kHz) were very high, and masked the vocalizations produced by the monk seals that were recorded in the 100–1000 Hz frequency bandwidth.

During the monitoring period, we observed a temporal pattern in both broadband (i.e., 20 Hz–5.5 kHz, Figure 5b) and octave-band levels (Figure 5c) that was driven primarily by the snapping shrimps (2000 Hz-band, Figure 5c). Acoustic levels tended to be lower during the day and higher at night (Figure 5b). The background noise level generated by these small crustaceans dominated the soundscape, as their snapping activity reached levels of up to 123 dB re 1 μPa (2 kHz band, Figure 5c) and was much higher than the noise generated by boats (125 Hz band, Figure 5c).

## 4. Discussion

The acoustic monitoring of an important reproductive area in north Evia Island indicated the presence of underwater vocalizations that we attributed to the Mediterranean monk seals. A minimum of twelve different individuals of all age and sex classes were identified in our study area, and the recordings in our study were made during the pupping season, a period during which pinnipeds are known to be highly vocal. In this context vocalizations are usually produced for territorial defense among males, mate selection, and mother–pup interactions [6,7].

Among the 18 different call types that we described, some showed a similar general acoustic structure to that described previously in in-air vocalizations of Mediterranean monk seals [28], such as barks and screams. Barks can be produced by both sexes and all age classes, whereas screams (long and short) are mostly produced by adults and juveniles [28]. Some of the other underwater call types recorded in our study, such as croaks, groans, growls, moans, and rumbles, were similar to the underwater calls that have been recorded in a male Hawaiian monk seal [32].

Some other call types recorded in our study were very similar in their general acoustic characteristics to underwater vocalizations described in other phocids. The short, loud, and repetitive calls recorded in our study, such as claps and knocks, were similar to the C and P sounds produced by Weddell seals *Leptonychotes weddellii* [42], to knocks and clicks produced by ice-breeding grey seals *Halichoerus grypus* [43], to the percussive non-vocal sound produced by grey seals using their flippers [44], or the knocks and taps produced by the Atlantic walrus *Odobenus rosmarus rosmarus* during the breeding season [45]. The cry described in the present study was similar to the roar produced by grey seals [43]. Some other call types, especially the very low-pitched calls, such as whines, wops, and woms, were more difficult to compare to other known vocalizations, as the quality of spectrograms found in publications is often poor and makes comparisons difficult. The only other marine mammals present in the area are bottlenose (*Tursiops truncatus*) and common dolphins (*Delphinus delphis*). A fin whale (*Balaenoptera physalus*) has also been recorded once in the area [2]. Our recordings did not show any acoustic similarities with the vocalizations of these species [46,47,48,49].

Given the general acoustic similarities (e.g., duration, spectral features, fundamental frequency) of our recordings with vocalizations known to be produced by the two extant monk seal species, either in-air or under water and with already-described underwater vocalizations of other pinnipeds, we believe that these calls were likely produced by the Mediterranean monk seals living in our study area. The vocal repertoire of the Mediterranean monk seal that we have established in our study needs to be confirmed in other areas of the species’ range by performing additional underwater recordings using passive acoustics. Finally, underwater recordings could be made on individuals in rehabilitation [29].

Random Forest analysis validated the results of our initial aural–visual classification, as all correct classification rates were greater than those obtained by chance. However, some call types were misclassified, suggesting that some call types could be potentially grouped together. For example, within the harmonic call category, croaks and cries were often confused with barks, and gloogloos with groans. Croaks, cries, and barks showed similar spectral characteristics (e.g., energy quartiles) and had fundamental frequency values that were within the same range. However, cries were, on average, much longer in duration than barks. Potentially, croaks, cries, and barks could be graded vocalizations and could explain such a misclassification. Similarly, gloogloos were misclassified as groans, as they shared the same spectral features and total duration, but groans tended to be lower-pitched than gloogloos (i.e., lower fundamental frequency values).

The masking effect of seal vocalizations by anthropogenic “noise” was detected in our recordings. Boat traffic in our study area was permanent, i.e., throughout the day and over the entire study period. This affected our study, as many vocalizations could not be measured and limited the frequency bandwidth at which we were able to perform our acoustic analysis. As a result, some low-frequency call types (e.g., croaks and woms) might have been masked by the low-frequency noise of boat traffic and therefore not analyzed, while other call types might not have been detected at all or with difficulty because of their low amplitude (e.g., moans). Given the effects that boat traffic has had on our ability to study the underwater vocalizations of the Mediterranean monk seal, we speculate that it is not unlikely that this noise might have negatively affected the species as well.

Vocal communication is essential for several marine species, such as pinnipeds, as it allows them to maintain social contact [8,50]. It is especially important for mothers and their pups in maintaining their social bond and in warning of potential dangers. It is also essential in ensuring breeding success by attracting mates and assessing rivals. If communication is masked or otherwise hindered, such vital functions cannot be ensured, thus potentially leading to lethal effects for the species [23]. Moreover, exposure to chronic anthropogenic noise can induce high levels of physiological stress, as in the case of North Atlantic right whales *Eubalaena glacialis* [51]. Physiological stress, as revealed by high levels of glucocorticoids, may lead to severe effects on health and reproduction [52,53].

If boat noise did indeed negatively affect the Mediterranean monk seals in our study area, then this is of particular conservation concern, given the endangered status of the species. This, in turn, will need to be taken into account in the conservation guidelines for the species [5], and relevant management measures need to be designed and effectively implemented.

### Management Implications

Replicating the research design of our study throughout the range of the species would be important, not only for further understanding the underwater vocal repertoire of the Mediterranean monk seal, but also for investigating the occurrence of geographical variations in the vocal repertoire of the species. Geographical variations in vocal repertoires have been detected in several phocids species, such as Weddell *Leptonychotes weddellii*, leopard *Hydrurga leptonyx*, harbor *Phoca vitulina*, and bearded seals *Erignathus barbatus*. For a review, see Charrier and Casey [7]. Variations in the vocal repertoire can occur not only at the repertoire level (e.g., new call types could be detected in new areas, or already-described call types might not be present in other areas) but also at the call type level, with variations, for example, of the acoustic characteristics (e.g., a call type might be found with a longer duration, higher- or lower-pitched).

Such passive acoustic monitoring research efforts could be paired/complemented by the use of acoustic tags placed on adult males and/or females that would not only enable the recording of their vocal repertoire but also associate it with behavioral aspects inferred through data from other sensors, such as video cameras, 3D accelerometers, and/or depth recorders, as has been done primarily with cetaceans [54,55,56]. Working at an individual level would be beneficial, as one could investigate individual vocal signatures that then could be used in the development of a classification algorithm to estimate the number of individuals from vocalizations collected during passive acoustic monitoring.

Information from such efforts would be important and function as baseline information in efforts to use the knowledge of the underwater vocal repertoire of the Mediterranean monk seal to set up acoustic monitoring schemes in locations that are known to be used by the species, but also in areas where the presence of the species is suspected/expected and has not been verified yet. Given the inherent difficulties involved in the visual monitoring of the species (i.e., low population numbers, secretive behavior, inaccessible habitat), passive underwater acoustic monitoring could develop into a reliable alternative for studying and protecting the endangered Mediterranean monk seal.

## 5. Conclusions

This study presents the first description of the underwater vocal sounds of the endangered Mediterranean monk seal. The vocal repertoire of the species is highly diverse, as 18 different call types divided into three main call categories (harmonic, noisy, and pulsative) were described. The findings of our study can be used to develop a long-term, acoustic monitoring scheme of (important) reproductive areas of the species, but also for identifying new potential areas of species occurrence.

The findings of our study also indicated high levels of anthropogenic noise in an important reproductive area of the Mediterranean monk seal, thus highlighting, for the first time in the species’ conservation history, ambient “noise” as a potential threat for this endangered species and the necessity for including this fact in effective conservation planning.

## Figures and Tables

**Table 1:** Acoustic characteristics of the 17 call types from the three main categories. For each variable, the sample size (n) of calls and the mean and SD values are presented.

Harmonic Calls	dur (s)	f0 (Hz)	excF (Hz)	Fmax (Hz)	Q25 (Hz)	Q50 (Hz)	Q75 (Hz)
Bark (*n* = 294)	0.418 ± 0.262	137 ± 37	45 ± 27	312 ± 190	275 ± 83	450 ± 134	687 ± 148
Croak (*n* = 32)	0.460 ± 0.147	96 ± 40	43 ± 29	282 ± 146	238 ± 86	366 ± 162	622 ± 406
Cry (*n* = 24)	1.386 ± 1.074	290 ± 150	95 ± 77	360 ± 170	272 ± 106	433 ± 118	699 ± 129
Gloo (*n* = 76)	0.350 ± 0.359	112 ± 29	74 ± 43	147 ± 79	153 ± 53	283 ± 109	490 ± 147
Gloogloo (*n* = 39)	0.555 ± 0.562	116 ± 28	--	193 ± 80	171 ± 37	288 ± 61	503 ± 119
Groan (*n* = 78)	0.500 ± 0.247	79 ± 36	--	174 ± 87	178 ± 58	287 ± 82	507 ± 289
Scream (*n* = 19)	0.408 ± 0.277	1207 ± 444	150 ± 111	1207 ± 444	--	--	--
Whines (*n* = 47)	0.663 ± 0.313	134 ± 45	51 ± 30	143 ± 41	--	--	--
Whoo (*n* = 65)	0.207 ± 0.068	439 ± 154	73 ± 48	461 ± 190	--	--	--
Wop (*n* = 23)	0.229 ± 0.069	297 ± 70	68 ± 30	406 ± 145	316 ± 59	486 ± 110	699 ± 94
Wom (*n* = 42)	0.303 ± 0.100	95 ± 30	54 ± 33	100 ± 44	117 ± 39	288 ± 157	632 ± 191
**Noisy calls**	**dur** **(s)**	**Fmax** **(Hz)**	**Q25** **(Hz)**	**Q50** **(Hz)**	**Q75** **(Hz)**		
Growl (*n* = 442)	1.136 ± 0.616	648 ± 412	542 ± 249	920 ± 388	1399 ± 662		
Hiccup (*n* = 210)	0.177 ± 0.042	395 ± 109	353 ± 82	422 ± 99	567 ± 120		
Squeak (*n* = 43)	0.155 ± 0.081	557 ± 147	422 ± 92	556 ± 92	690 ± 105		
**Pulsative calls**	**dur** **(s)**	**Fmax** **(Hz)**	**Q25** **(Hz)**	**Q50** **(Hz)**	**Q75** **(Hz)**	**PR** **(Hz)**	
Clap (*n* = 13)	5.103 ± 1.619	--	--	--	--	1.70 ± 0.16	
Knock (*n* = 226)	1.922 ± 1.828	699 ± 441	--	--	--	6.81 ± 1.43	
Rumble (*n* = 23)	1.062 ± 0.434	205 ± 65	215 ± 50	347 ± 97	571 ± 142	16.31 ± 3.5	

**Table 2:** Random Forest classification matrix for the 11 harmonic call types. The overall error rate was 20.7%. The last column indicates the classification error for each main call type.

	Bark	Croak	Cry	Gloo	Gloogloo	Groan	Scream	Whine	Whoo	Wom	Wop	Classification Error
bark	**271**	2	2	11	1	0	0	0	0	4	3	0.08
croak	15	**14**	0	2	0	1	0	0	0	0	0	0.56
cry	9	0	**12**	0	0	1	0	0	0	0	2	0.50
gloo	17	2	0	**44**	2	2	0	0	0	9	0	0.42
gloogloo	7	0	0	1	**19**	11	0	0	0	1	0	0.51
groan	9	0	0	0	8	**61**	0	0	0	0	0	0.22
scream	0	0	0	0	0	0	**15**	0	4	0	0	0.21
whine	0	0	0	0	0	0	0	**46**	1	0	0	0.02
whoo	0	0	0	0	0	0	1	2	**62**	0	0	0.05
wom	6	0	0	10	0	2	0	0	0	**24**	0	0.43
wop	4	0	1	0	0	0	0	0	0	0	**18**	0.22

**Table 3:** Random Forest classification matrix for the three noisy call types. The overall error rate was 4.6%. The last column indicates the classification error for each main call type.

	Growl	Hiccup	Squeak	Classification Error
growl	441	1	0	0.002
hiccup	0	199	11	0.052
squeak	1	19	23	0.465

**Table 4:** Random Forest classification matrix for the three pulsative call types. The overall error rate was 1.54%. The last column indicates the classification error for each main call type.

	Clap	Knock	Rumble	Classification Error
clap	13	0	0	0
knock	0	222	2	0.009
rumble	0	2	21	0.086

## Data Availability

The data presented in this study are available in the Supplementary Materials.

## Supplementary Materials

The following supporting information can be downloaded at: https://www.mdpi.com/article/10.3390/ani13061048/s1, Table S1: Acoustic analysis of all call types. Sounds samples for each call type presented in Figure 1, Figure 2 and Figure 3.
